# What merits greater scholarly attention in international business?

**DOI:** 10.1057/s41267-022-00539-1

**Published:** 2022-07-01

**Authors:** Birgitte Grøgaard, Michael A. Sartor, Linda Rademaker

**Affiliations:** 1 Department of Strategy and Entrepreneurship, BI Norwegian Business School, Nydalsveien 37, 0484 Oslo, Norway; 2 Smith School of Business, Queen’s University, 143 Union Street, Kingston, ON K7L 3N6 Canada

**Keywords:** international business research agenda, MNEs, international business theory, future directions

## Abstract

Scholarly efforts to propose future directions for international business (IB) research have generated a timely and extensive inventory of potentially interesting areas of research. We supplement this line of inquiry by suggesting that an additional layer of scrutiny could be beneficial when advocating in favor of giving more attention to particular research realms. Specifically, we advance several guiding principles that will help IB scholars assess which research areas merit greater scholarly attention, based on their potential importance and impact. We distinguish between (1) research in new or underdeveloped research domains, where salience, urgency, and actionability are critical elements, and (2) new research in relatively well-established domains, where scholars may contribute to changing the theoretical conversations taking place in IB.

## Résumé

Les efforts scientifiques visant à proposer de futures directions de recherche en affaires internationales (International Business – IB) ont permis de dresser un inventaire complet et opportun de domaines de recherche potentiellement intéressants. Pour compléter les efforts précédents, nous suggérons qu'un niveau supplémentaire d’examen rigoureux et complet pourrait être bénéfique lorsqu'il s'agit de plaider en faveur d'une plus grande attention à des domaines de recherche particuliers. Plus précisément, nous élaborons plusieurs principes directeurs susceptibles d’aider les chercheurs en IB à évaluer quels domaines de recherche méritent une plus grande attention scientifique, en fonction de leurs importance et impact potentiels. Nous faisons la distinction entre (a) la recherche dans des domaines nouveaux ou sous-développés, où la saillance, l'urgence et l’actionnabilité sont les éléments essentiels, et (b) la recherche nouvelle dans des domaines relativement bien établis où les chercheurs peuvent contribuer à modifier les conversations théoriques ayant lieu dans le domaine de l’IB.

## Resumen

Los esfuerzos académicos para proponer direcciones futuras para la investigación en negocios internacionales han generado un inventario oportuno y extenso de las áreas de investigación potencialmente interesantes. Complementamos esta línea de investigación proponiendo una capa adicional de indagación que podría ser beneficiosa cuando se fomenta a favor de darle más atención a determinados ámbitos de investigación. Específicamente, proponemos varios criterios orientadores que van a ayudar a los académicos de negocios internacionales a evaluar cuales áreas de investigación merecen una mayor atención académica, en función de su importancia e impacto potenciales. Distinguimos entre (a) la investigación en ámbitos de investigación nuevos o poco desarrollados, en los cuales la prominencia, urgencia y posibilidad de acción son elementos críticos, y (b) nueva investigación en dominios relativamente bien establecidos, en donde los académicos pueden contribuir a cambiar las conversaciones teóricas que tienen lugar en negocios internacionales.

## Resumo

Esforços acadêmicos para propor direções futuras para a pesquisa em negócios internacionais (IB) geraram um acervo oportuno e extenso de áreas de pesquisa potencialmente interessantes. Complementamos essa linha de investigação sugerindo que uma camada adicional de escrutínio poderia ser benéfica ao defender a concessão de mais atenção a específicos domínios de pesquisa. Especificamente, avançamos vários princípios norteadores que ajudarão acadêmicos de IB a avaliar quais áreas de pesquisa merecem maior atenção acadêmica, com base em sua potencial importância e impacto. Distinguimos entre (a) pesquisa em domínios de pesquisa novos ou subdesenvolvidos, onde saliência, urgência e capacidade de ação são elementos críticos, e (b) novas pesquisas em domínios relativamente bem estabelecidos, em que acadêmicos podem contribuir para mudar as conversas teóricas que ocorrem em IB.

## Abstract (zh)

为国际商务 (IB) 研究提出未来方向的学术努力已经产生了一个及时而广泛的潜在有趣的研究领域的清单。我们通过建议在提倡对特定研究领域给予更多关注时进行额外层面的审视会可能会有益, 从而对这一努力进行了补充。具体来说, 我们提出了几项指导原则, 这些原则将帮助 IB 学者根据研究领域潜在的重要性和影响力来评估哪些研究领域值得给予更多的学术关注。我们区分了 (a) 在新的或不发达的研究领域的研究, 其中显著性, 紧迫性和可行动性是关键要素, 以及 (b) 在相对成熟的领域中的新研究, 其中学者可能有助于改变正在IB里发生的理论对话。

## INTRODUCTION

Advances in international business (IB) theory, the shifting tides of the global political economy, the growing prominence of grand societal challenges, and the relentless march of a worldwide pandemic are just a few of the developments that have been motivating scholars to reflect on and propose future directions for IB research (including, among others, Buckley, Doh, & Benischke, 2017; Ghauri, Strange, & Cooke, 2021; Lundan, 2018). Needless to say, these efforts continue to exert a positive impact on IB scholarship, and have generated a timely and extensive inventory of both underexplored and newly emerging research domains relevant to multinational enterprises (MNEs). This work is already stimulating a robust research stream that is advancing understanding in both new and established research domains. In their Point article, Arikan and Shenkar (2021) add to this stream by offering suggestions for what to cover *more* in international business to “deepen and strengthen the field’s reach and impact” They highlight several interesting areas for future research, and we commend their work as our academy debates the future of IB.

In this Counterpoint, we endeavor to supplement this line of inquiry, by suggesting that an additional layer of scrutiny could be beneficial when advocating in favor of giving more attention to particular research areas. We are especially concerned with how IB scholars can sustain or extend the relevance of IB as an academic research discipline. More specifically, the question that motivates our work is this: "How do we assess what merits greater scholarly attention in IB research?" In answering this question, rather than highlighting additional phenomena and contexts that are under-researched, we advance several guiding principles that are intended to aid scholars in assessing the merit of a proposed study. To do so, we organize our guiding principles along two key dimensions: (1) scholarship focused on new or underexplored research domains, and (2) scholarship focused on well-established research domains. Table 1 provides an overview of our six guiding principles. While these guiding principles may be particularly helpful to IB researchers, they may also be useful to scholars in other research fields.

**Table 1:** Guiding principles for assessing the importance and impact of a proposed study

	Principle	Description	Questions to ask
For studies in new or underexplored research domains	1. Salience for firms, societies, and/or policymakers	Addressing issues that are important to firms, societies, and/or policymakers. Important research “can positively influence individuals and groups within and outside organizations…[and] advance theories that influence our society or resolve important debates” (Tihanyi, 2020: 329)	To what extent does the study address current and/or future challenges? To whom are these issues important? Why are these issues important? To what extent does the study capture the “bigger picture” or external dynamics?
	2. Urgency of the phenomenon	Addressing issues that are current or likely to become important in the (near) future. This enhances the potential impact of studies	To what extent does the study address issues that are perceived as urgent by the target audience? Are these issues likely to be(come) important in the future?
	3. Actionability of the findings	Employing an action-oriented perspective towards proposed research. Detailing how findings can be acted upon by the study’s audience. Studies that can be acted upon are more likely to make a meaningful impact	Who is/are the audience(s) of the study? How can the potential outcomes be acted upon by the audience? Is it possible to clearly describe the potential course(s) of action emerging from the study?
For studies in established research domains	4. Reconciling equivocal research streams	Addressing ongoing debates or equivocal findings in the literature. The extent to which a study is able to resolve ongoing debates in the literature may influence the degree to which it impacts subsequent theoretical discourse	What is the puzzle generated by the accumulated findings in the extant literature? How can the proposed study address this stalemate? How will this extend theory or change the theoretical conversation?
	5. Fostering interdisciplinary exchanges and engagement among scholars	Engaging with other disciplines. Studies that engage meaningfully with other disciplines are more likely to have a greater impact, because they increase scholars’ exposure to new ideas, theoretical perspectives, and empirical methods, which can be used to foster new insights and solutions to practical problems facing the target audience	To what extent does the proposed study engage with other disciplines? How may the topic of the study be relevant for other disciplines? To what extent could the proposed study benefit from the ideas, theories, and/ or methods from other academic fields to foster new insights and solutions?
	6. Elaborating and testing theoretical mechanisms	Elucidating theoretical mechanisms. These focus on the “how” question in research. Studies that elucidate theoretical mechanisms are important to the development of the field by adding precision to our theoretical and empirical conceptualizations	What are the theoretical explanations for the relationship between an independent and a dependent variable? How has this mechanism been studied in the literature? To what extent have prior studies tested the plausibility of the mechanism(s) that have been theorized regarding the relationship between two constructs? How can the proposed study explicate the mechanism(s) of interest?

Scholarly efforts to propose future directions for international business (IB) research have generated a timely and extensive inventory of potentially interesting areas of research. We supplement this line of inquiry by suggesting that an additional layer of scrutiny could be beneficial when advocating in favor of giving more attention to particular research realms. Specifically, we advance several guiding principles that will help IB scholars assess which research areas merit greater scholarly attention, based on their potential importance and impact. We distinguish between (1) research in new or underdeveloped research domains, where salience, urgency, and actionability are critical elements, and (2) new research in relatively well-established domains, where scholars may contribute to changing the theoretical conversations taking place in IB

## NEW OR UNDEREXPLORED RESEARCH DOMAINS: ASSESSING IMPORTANCE AND IMPACT

The IB field is currently at a crossroads. Concerns have persisted that the field may not be devoting sufficient attention to the important big questions (Buckley, 2002; Doh, 2017). As academics, we have the privilege to dive deep into specific areas of research that *interest* us. Indeed, the broader management field has successfully motivated scholars to pursue interesting research questions through helpful editorials (e.g., Grant & Pollock, 2011) and prestigious research awards (e.g., the “That’s Interesting!” award from Aalto University). These efforts have encouraged researchers to think outside the box and to advance new ideas, fostering many notable contributions to the field. However, we recommend taking a step back and reconsidering what merits greater scholarly attention in IB research before proceeding. Consistent with the ongoing debate on the future of management research (Tihanyi, 2020), we maintain that *interesting* IB research may differ from *important* and *impactful* research.

Our intent is not to add to this debate by generating yet another list of research possibilities; rather, we argue that proposed research priorities in IB must have more to motivate them than simply being “interesting.” Thus, the intention behind our guiding principles is to encourage scholars to specifically consider the potential importance and impact for scholars, firms, societies, and/or policymakers at the preliminary stages of their research projects. To satisfy this objective, we maintain that IB researchers focusing on new or underexplored research domains should consider the salience, urgency, and actionability of their proposed work. We elaborate on these guiding principles below.

### Guiding Principle #1: Salience for Firms, Societies, and/or Policymakers

The salience of research for practice has been a topic of discussion for several decades (Collinson, 2017; Daniels, 1991; Teagarden, Von Glinow, & Mellahi, 2018). Scholars are inherently interested in balancing rigor and relevance as they design research studies (Teagarden et al., 2018; Tsui, 2019). Notably, the IB field had a history of focusing on research based on managerial insights (e.g., Bartlett & Ghoshal, 1989; Johanson & Vahlne, 1977), but contemporary scholarship has increasingly distanced itself from “real-world” phenomena (Buckley et al., 2017); further, managerial relevance has declined over time as the field has matured (Doh, 2015).

To enhance the salience of their proposed research, scholars should engage with stakeholders to identify issues that may be pertinent to businesses or societies (Drnevich, Mahoney, & Schendel, 2020). Consistent with our perspective, Tihanyi (2020: 329) observes that

important management research can positively influence individuals and groups within and outside organizations, has the potential to improve living conditions in societies, shows ways to build or strengthen ties across nations, or provides findings that consider the interests of future generations…[and] advance[s] theories that influence our society or resolve important debates.

Building on Arikan and Shenkar (2021), we maintain that pertinent audiences include academic researchers, IB students, executives and middle managers in firms and NGOs, and local or global policymakers. For example, following the tragic deaths during the 2013 Dhaka garment factory collapse, Narula (2019) examines the complexities of control in global value chains. He points to stakeholder expectations that MNEs take “full chain” responsibility to reduce the exploitation of workers in countries with lower labor standards. This study contributes to an important debate in the IB literature on the link between ownership and MNE control and coordination, one that has an impact on the challenges facing efficiency-seeking MNEs and the societies in which they locate activities.

IB researchers have also been encouraged to address other international dilemmas, given that they relate to topics under scrutiny in the IB domain, and, by definition, span multiple countries. This has generated a plethora of research questions in IB, such as, how MNEs influence, and are influenced by, the long-term energy transition. In their study of the European solar industry, Georgallis, Albino-Pimentel, and Kondratenko (2021) show how policymakers can positively impact MNE location decisions, thereby contributing to the shift towards renewable energy. Similarly, Patnaik (2020) shows that MNEs react differently than domestic firms to regulatory initiatives, and thus differ in the ways in which they are able to benefit from and mitigate the costs associated with emissions trading policies. These studies illustrate research that is salient to multiple stakeholders, including firms, societies, and policymakers.

There have been numerous calls for scholars to be more mindful of emerging changes in the global business environment, (e.g., Buckley & Lessard, 2005; Doh, 2015; Ghauri et al., 2021). Arikan and Shenkar (2021) suggest clustering the main building blocks of the field into its audience, locations, environments, history, and interactions. They also propose that these building blocks should guide IB’s “reach, scope, focus, and attention” (Arikan & Shenkar, 2021: 3). While this approach may yield research that can be characterized as interesting, we believe that scholars must also engage in the more fundamental assessment of whether the research is important and impactful. More specifically, when calling attention to phenomena or contexts that are characterized as under-researched, scholars must also assess the potential of the research to address the “bigger picture” of current and future challenges faced by firms, societies, or policymakers.

### Guiding Principle #2: Urgency of a Research Topic

The importance and impact of a research topic is frequently linked to its perceived urgency. Recent studies have pointed to the need to recognize significant changes and dynamics in the IB context, such as geopolitical shifts, the digitalization of firms and markets, the renegotiation of regional integration schemes, and the heightened worldwide engagement with the UN’s Sustainable Development Goals (for a thorough overview, see, e.g., Ghauri et al., 2021). These changes not only create new business and policy challenges but also pose new questions for established IB theories related to firm boundaries, roles, and responsibilities as MNEs evolve (Narula, Asmussen, Chi, & Kundu, 2019).

For some phenomena, such as climate change, the sense of urgency is readily apparent. The Intergovernmental Panel on Climate Change (IPCC) recently reported a dismal outlook for societies unless firms and policymakers take immediate action to limit human-induced global warming (IPCC, 2021). Similarly, the COVID-19 pandemic has exposed the vulnerabilities of global value chains (Gereffi, 2020). These current dynamics create opportunities for scholars to engage with topics that are in urgent need of attention. For example, Verbeke and Yuan (2021) re-examine the risks and benefits for MNEs when deciding whether to internalize or decouple value chain activities following the pandemic. The list of urgent challenges continues to grow; we have only scratched the surface by highlighting the above examples.

Other issues require deeper thought and reflection with respect to their urgency. Assessing which trends will have an impact in the future is not an easy task, but nevertheless warrants significant attention for the process of proposing areas for future research. For example, Geddes, Nuttall, and Parekh (2020) argue for more attention to stakeholder engagement, based on their findings that over 60% of CEOs identify external stakeholder engagement as one of their top three priorities, although the majority of firms surveyed struggled to align their business with the interests of their various stakeholders. In their studies on shared value, Porter and Kramer (2006, 2011) explore how companies can align firm and stakeholder interests to generate win––win solutions. While their approach clearly does not prescribe solutions to societal problems such as poverty, or global issues such as climate change, it does provide managers with tools for engaging in urgent and essential stakeholder conversations to address immediate problems facing firms and the local communities where they operate (Dembek, Singh, & Bhakoo, 2016).

### Guiding Principle #3: Actionability of the Findings

In addition to assessing the salience and urgency of the planned research, scholars should consider how their proposed study can be oriented for the target audience to take action. We agree with Arikan and Shenkar (2021) that it is important for IB scholars to consider who our audience is. Numerous calls have been made for research that is relevant and actionable for managers (Bartunek & Egri, 2012; Doh, 2015). Ideally, research findings should enable the target audience to make better decisions. Consequently, research that offers insights into how to improve decision-making has greater potential to make an impact outside the scholarly community. This may sound self-evident, but a significant portion of published research in IB tends to overlook the managerial implications. Instead, it is common to find only a cursory consideration of a study’s practical implications in the discussion section of published IB papers. Although a growing number of academic journals are prompting researchers to include a section on managerial and/or policy implications, these sections often seem to be afterthoughts rather than core drivers of the research focus and design (Bartunek & Rynes, 2010; Joullié & Gould, 2021). Moreover, given the need to convey theoretical contributions in their work, many researchers prioritize conceptual advances over practical implications (Hambrick, 2007). Consequently, most published research seems to be primarily intended for an academic audience. It is not surprising then that the practical implications in IB research are often limited or not fully communicated.

We recognize that it can be challenging to clearly describe how a study’s target audience can act upon its findings. As a result, the language employed often suggests that managers or policymakers should “take something into account.” However, if studies have defined an audience, and address a topic important to that audience, we should also be able to describe proposed actions. In their editorial on action-based dynamic capabilities for IB, Zahra, Petricevic, and Luo (2022) suggest that attention to underlying processes and mechanisms is one approach to make research actionable. Further, it would be helpful to describe the circumstances under which the proposed actions apply. Contextualization not only facilitates the ability to act upon a study’s findings but also helps to understand their generalizability (Teagarden et al., 2018).

## ESTABLISHED RESEARCH DOMAINS: ASSESSING POTENTIAL THEORETICAL CONTRIBUTIONS

Cultivating new insights into IB that are both important and impactful to theory and practice can clearly be achieved by executing salient, urgent, and actionable research in underexplored and newly emerging research domains. However, we propose that there is also still much work to be done, and considerable value inherent, in pushing the boundaries of research further with respect to well-established areas. There are three ways, at least, that IB scholars can change the conversation or re-position/re-frame well-established research domains, by engaging in research that: (1) reconciles streams of prior equivocal research findings; (2) fosters interdisciplinary exchanges and engagement among scholars; and/or (3) elaborates and tests theoretical mechanisms.

### Guiding Principle #4: Reconciling Equivocal Research Streams

IB scholars are adept at defining and executing comprehensive research agendas. As just one example, research pertaining to the role of home- and host-market institutions upon the strategies and performance of foreign-investing MNEs has generated a substantial body of IB literature during the past few decades (Marano, Arregle, Hitt, Spadafora, & van Essen, 2016; Tang & Buckley, 2020). In fact, the research questions within this domain continue to be pressing. However, a closer examination of the accumulated work reveals that contradictory empirical findings persist regarding the impact of distinct types of institutions. While calls for increased empirical rigor (Meyer, van Witteloostuijn, & Beugelsdijk, 2017) play an integral role in efforts to reconcile equivocal research findings in IB research domains, an important opportunity also exists for scholars to contribute at a theoretical level to these efforts by refining the conceptualization of constructs in ways that enhance our understanding of phenomena.

Several recent examples illustrate how this can be achieved. For instance, Holmes Jr., Miller, Hitt, and Salmador (2013) synthesized precepts from organizational institutionalism and institutional economics to disaggregate the formal institutions construct into three sub-types: regulatory institutions, political institutions, and economic institutions Their empirical analyses of the impact of these three disparate categories of formal institutions suggested that each exerted distinct effects on inward foreign direct investment (FDI). Similarly, Estrin and Prevezer (2011) leveraged Helmke and Levitsky’s (2004) four types of informal institutions – complementary, accommodating, competing, and substitutive – to explore the role of informal institutions in corporate governance in several emerging market countries. Both Holmes Jr. et al. (2013) and Estrin and Prevezer (2011) make unique contributions that help to reconcile conflicting research findings relevant to the role of national institutions. While Holmes Jr. et al. (2013) provide a more fine-grained understanding of the relationship between the country-level institutional environment and foreign inward investment behavior, Estrin and Prevezer’s (2011: 43) work helps to organize “the disparate literature on formal and informal institutions, and clarifies some of the contrasting relationships found in emerging economies as to how these institutions interact and affect governance.” Taken together, their work provides a theoretical foundation upon which scholars can begin to disentangle equivocal research findings pertinent to the impact of institutions on other phenomena in IB research, an opportunity that we discuss further below.

We share Arikan and Shenkar’s (2021) general interest in encouraging researchers to devote greater scholarly attention to the contribution of neglected firms, such as small-to-medium-sized enterprises (SMEs), to global FDI flows, among other outcomes. However, our guiding principles urge IB scholars to reflect upon how their research concerned with SMEs might serve to change the scholarly conversation about the internationalization of SMEs. Building upon our earlier observation that research focused on the interrelationship between institutions and the investment behavior of firms has been characterized by streams of conflicting findings, IB researchers have noted the accumulation of divergent research findings about the relationship between regulative (or formal) institutions and the governance of SMEs’ subsidiary investments (Bruneel & De Cock, 2016; Laufs & Schwens, 2014). While some scholars have concluded that weak formal institutions prompt SMEs to invest abroad through joint ventures, others have found that full ownership will be employed, and still others have determined that formal institutions in the foreign host market have no impact on the governance choice. To untangle these findings, Laufs and Schwens (2014: 1110) suggested that “it is important to reflect on the boundary conditions of existing theory and to examine potential sources of variation.” Leveraging the work of both Holmes Jr. et al. (2013) and Estrin and Prevezer (2011) may equip scholars to reconcile the equivocal research findings in this domain. Alternatively, researchers might also choose to elaborate typologies of formal and informal institutions that are even more relevant to the experience of SMEs.

Efforts to reconcile streams of equivocal research findings can change the conversation and make important contributions to IB research in two key ways. First, a more fine-grained conceptualization of extant constructs contributes to theoretical extensions which expand our “cumulative body of knowledge” (Meyer & Peng, 2016: 14). In this regard, efforts to reconcile divergent findings in relatively mature research domains serves to enrich theory by expanding the network of constructs and conceptual tenets embedded within a given theoretical domain. Second, refining the conceptualization of a construct can help to communicate theoretical boundary conditions regarding the impact of the construct upon a specified dependent variable (Suddaby, 2010).

### Guiding Principle #5: Fostering Interdisciplinary Exchanges and Engagement among Scholars

Another important opportunity for IB scholars to change the conversation, both within the IB domain and beyond, exists in developing research that can foster authentic, robust, and enduring discourse with adjacent academic disciplines, such as law, finance, political science, economics, sociology, and geography, among others. IB scholars have consistently advocated for an interdisciplinary approach to investigate phenomena (Cantwell, Piepenbrink, & Shukla, 2014; Cheng, Henisz, Roth, & Swaminathan, 2009; Dunning, 1989). We maintain that research that integrates “ideas and/or methods from two or more disciplines…[to] produce something new and useful (in either solving a problem or advancing fundamental understanding)…[that] could not have been obtained by…one single discipline alone” (Cheng et al., 2009: 1071) plays an important role in sustaining and extending the relevance of IB as an academic research discipline.

First, interdisciplinary research helps to increase IB scholars’ exposure to new ideas, theoretical perspectives, and empirical methods, which, in turn, can be used to foster new insights and solutions to the practical problems that confront MNEs and their managers in foreign markets (Cheng, Birkinshaw, Lessard, & Thomas, 2014). Second, engaging in interdisciplinary scholarship could provide an opportunity to broaden the impact of IB, because engaging in research with other academic disciplines holds the potential to engender social value (Currie, Davies, & Ferlie, 2016). Despite these potential benefits, Buckley et al., (2017: 1046) have observed that “while IB scholars tend to embrace interdisciplinary perspectives initially when studying and explaining relatively new phenomena, as these research streams mature, scholars appear to become more inward-looking and self-referential.”

This is regrettable, because interdisciplinary exchanges hold the potential to change conversations in IB research and reframe how we look at more established domains. As examples, IB scholars have succeeded in fostering more enduring interdisciplinary discourse with finance scholars on issues related to global corporate governance (Cumming & Walz, 2010; Cumming, Filatotchev, Knill, Reeb, & Senbet, 2017; Cumming, Siegel, & Wright, 2007), economic geographers on matters of innovation and location choice (Beugelsdijk & Mudambi, 2013; Beugelsdijk, McCann, & Mudambi, 2010; McCann & Mudambi, 2005), and business ethicists in efforts to extend the application of integrative social contracts theory to practice (Husted & Allen, 2006; Spicer, 2009; Spicer, Dunfee, & Bailey, 2004).

Many research areas lend themselves to interdisciplinary research. For example, phenomena pertaining to corporate social performance/responsibility, as well as phenomena that are related to initiatives like UN’s Sustainable Development Goals, are providing particularly fertile terrain for IB researchers to engage in interdisciplinary scholarship. Indeed, continuing calls for increased attention to such phenomena and their interrelationship with MNEs are emanating from the fields of IB (Van Tulder, Rodrigues, Mirza, & Sexsmith, 2021), business ethics (Bowie, 2019), economics (Halkos & Nomikos, 2021), political science (Bull & Miklian, 2019), and law (Sjåfjell, 2018; Sjåfjell & Taylor, 2019).

### Guiding Principle #6: Elaborating and Testing Theoretical Mechanisms

Conceptual mechanisms are “underlying theoretical processes (or reasons) for certain proposed effects” (Andersson, Cuervo-Cazurra, & Nielsen, 2014: 1070). During the past decade, IB scholars have engaged in more pronounced efforts to detail the theoretical mechanisms underpinning relationships between constructs in a phenomenon’s ecosystem (Thomas, Cuervo-Cazurra, & Brannen, 2011). Nevertheless, Buckley et al., (2017: 1048) observed that the literature in some core phenomenon-driven IB research areas still offers “little insight into the mechanisms by which” relationships occur. Consequently, the growing demand for mechanism-based theorizing and the testing of mechanisms provides two important opportunities for IB scholars to enhance the ongoing conversations about well-established phenomena in IB research.

First, prior published work may present statistically significant empirical results as evidence that supports a hypothesized relationship between two constructs. However, theoretical advances demand more than this. If researchers do not also provide a theoretical explanation regarding *how* the dependent variable or outcome arises from an independent variable, then a more comprehensive account of the nature of the relationship (“the mechanism”) is still needed (Whetten, 1989). Second, prior published work may attempt to describe the proposed theoretical mechanisms that underpin a particular relationship between constructs. However, Bromiley and Johnson (2005: 17–18) reminded scholars that the quality of mechanism-based explanations depends upon “the correctness of the preconditions, the generality of the mechanisms and the accuracy of the predictions…A good…test of an explanation should test all three as directly as possible.” While an in-depth survey of suitable methodological approaches is outside the scope of this Counterpoint, the theoretical mechanisms through which outcomes arise can be identified using either qualitative or quantitative methods (Reeb, Sakakibara, & Mahmood, 2012: 217). For instance, Clougherty and Skousen (2021) empirically demonstrate that, while the literature on cross-border M&As has implicitly assumed that most MNE activity is driven by efficiency motivations, market-power considerations actually account for one-third of MNE activity. We encourage further scholarly discourse that explores appropriate methodological approaches in relation to testing theoretical mechanisms.

We believe that mechanism-based research represents critically important opportunities for IB scholarship to contribute to the continued evolution and accumulation of knowledge in more well-established research domains, when it (1) provides theoretical explanations about *how* the dependent variable arises from an independent variable, or (2) tests the plausibility of the mechanism(s) that have been theorized regarding the relationship between two constructs. Two reasons account for why this work matters. First, mechanism-based theorizing deepens our understanding of the relationships identified between constructs, providing a more comprehensive account of the processes that operate within a phenomenon’s ecosystem (Bromiley & Johnson, 2005). Second, given that distinct theoretical perspectives postulate the operation of different conceptual mechanisms between constructs, testing theorized mechanisms helps to elucidate which theories offer more plausible explanations for observed phenomena (Bromiley & Johnson, 2005).

## CONCLUSIONS

In this Counterpoint, we have presented six guiding principles to help IB scholars assess what merits greater attention in IB research. We have organized these guiding principles along two dimensions: (1) scholarship focused on new or underexplored research domains; and (2) scholarship focused on well-established research domains. Our guiding principles are intended to enhance efforts to select research projects, to motivate studies, and to assess the potential contributions of the proposed work. Given the ongoing debate in IB about relevance and the future of the field, we hope these guiding principles will help spur research that addresses these concerns, by prioritizing topics that are important, impactful, and have the potential to change the conversation in IB. One of the challenges associated with advancing guiding principles about areas worthy of more research (in both established and newly emerging domains) is the need to ensure that the principles are parsimonious, yet concrete enough that they can be implemented.

Ultimately, our guiding principles have been developed to provide a first layer of scrutiny to assess a proposed study’s importance and potential impact, after which researchers can assess the appropriate research design, to craft a theoretically sound and rigorous research project. As such, we believe that our guiding principles will be particularly useful to scholars in the preliminary stages of a research project. We are not suggesting that IB scholars should be expected to adhere to all of these guiding principles simultaneously. At the same time, the principles are not mutually exclusive. Instead, their applicability will vary, depending on the intended audience and the nature of the research domain.

Unquestionably, studies motivated by macro- or micro-level developments in the MNE’s context should attend to our first three guiding principles of salience, urgency, and actionability. In essence, this challenges scholars to engage more, whether directly or indirectly, with their target audiences. Multiple ways exist to engage with audiences. First, a straightforward and low-threshold approach is to link research questions to news headlines and issues currently garnering public attention. This can signal both salience and urgency, as exemplified in studies on labor conditions in developing countries (Narula, 2019) and post-pandemic value chain reliability (Gereffi, 2020). Second, scholars can collect primary data to better assess their target audiences’ thoughts about the potential importance and impact of proposed researched topics. Primary data sources, such as managerial interviews and internal company documents, offer valuable insights into internal processes and decisions that help scholars identify challenges that firms consider salient or urgent.

Third, scholars can engage in the process of co-creation (Grodal, Anteby, & Holm, 2021; Prescott & Filatotchev, 2021; Sharma & Bansal, 2020), whereby researchers and practitioners work together to address potentially important and impactful research. Finally, many scholars have ample opportunity to engage in conversations with target audiences through classroom interactions, industry conferences, and university-wide engagement with alumni, local communities, and other stakeholders, all of which provide opportunities for enhancing both the relevance and rigor of our research (Tushman, O'Reilly, Fenollosa, Kleinbaum, & McGrath, 2007). In fact, prior to writing this Counterpoint, we engaged in informal conversations with senior international executives who shared the challenges that they believe are facing firms and industries worldwide. These interactions confirmed for us that the target audiences for our work are ready and willing to engage with scholars to help identify important research opportunities that can potentially make a meaningful impact upon both theory and practice.

In contrast, research along the second dimension, which lies in more established domains, may primarily aim to synthesize research or to add nuance to extant theoretical explanations. For example, review studies that survey the accumulated academic literature frequently identify research areas in which there are conflicting findings, a need to clarify mechanisms, or a shortage of interdisciplinary discourse. Consequently, prior to embarking on proposed research in well-established domains, scholars should consider our last three guiding principles. This entails assessing whether the work holds the potential to change or enhance the theoretical conversations taking place in IB, by reconciling equivocal research streams, fostering interdisciplinary exchange, or elaborating or testing theoretical mechanisms. Nonetheless, we are not suggesting that the scholarly community is the only audience for research in more established domains. For example, the importance and impact of studies elaborating and testing mechanisms could also offer new insights to a broader range of audiences, including managers and policymakers. Moreover, opportunities may arise for researchers to test a study’s theoretical mechanisms by engaging more deeply with its target audience, thereby enhancing the actionability of a study. Our aim is to encourage researchers to use these guidelines as a way to assess the potential for making an impact, both from a theoretical and practical stance.

In closing, the goal of our Counterpoint has not been to generate another list of potentially interesting research phenomena or questions that need more attention in IB research. Instead, our motivation has been to challenge IB scholars to engage in an added layer of scrutiny, one that highlights the importance and potential impact of their proposed work when advocating in favor of giving more attention to particular research areas. Given our interest in how IB scholars can sustain or extend the relevance of IB as an academic research discipline, we encourage our community to apply our six guiding principles to their proposed research. Furthermore, we hope others will build on this list of guiding principles over time, to advance IB scholarship. Every research project has a life cycle. Our guiding principles are primarily intended to support scholars during the preliminary stages of their research projects, when deciding upon the scope and focus. Nevertheless, we anticipate that these guiding principles will also serve as a useful touchstone for scholars to revisit as their research projects progress, particularly when articulating the ongoing importance and impact of their studies.
